# Catastrophic health expenditure and 12-month mortality associated with cancer in Southeast Asia: results from a longitudinal study in eight countries

**DOI:** 10.1186/s12916-015-0433-1

**Published:** 2015-08-18

**Authors:** The ACTION Study Group

**Affiliations:** The George Institute for Global Health, Level 10, King George V Building, 83–117 Missenden Road, Camperdown, NSW 2050 Australia

## Abstract

**Background:**

One of the biggest obstacles to developing policies in cancer care in Southeast Asia is lack of reliable data on disease burden and economic consequences. In 2012, we instigated a study of new cancer patients in the Association of Southeast Asian Nations (ASEAN) region – the Asean CosTs In ONcology (ACTION) study – to assess the economic impact of cancer.

**Methods:**

The ACTION study is a prospective longitudinal study of 9,513 consecutively recruited adult patients with an initial diagnosis of cancer. Twelve months after diagnosis, we recorded death and household financial catastrophe (out-of-pocket medical costs exceeding 30 % of annual household income). We assessed the effect on these two outcomes of a range of socio-demographic, clinical, and economic predictors using a multinomial regression model.

**Results:**

The mean age of participants was 52 years; 64 % were women. A year after diagnosis, 29 % had died, 48 % experienced financial catastrophe, and just 23 % were alive with no financial catastrophe. The risk of dying from cancer and facing catastrophic payments was associated with clinical variables, such as a more advanced disease stage at diagnosis, and socioeconomic status pre-diagnosis. Participants in the low income category within each country had significantly higher odds of financial catastrophe (odds ratio, 5.86; 95 % confidence interval, 4.76–7.23) and death (5.52; 4.34–7.02) than participants with high income. Those without insurance were also more likely to experience financial catastrophe (1.27; 1.05–1.52) and die (1.51; 1.21–1.88) than participants with insurance.

**Conclusions:**

A cancer diagnosis in Southeast Asia is potentially disastrous, with over 75 % of patients experiencing death or financial catastrophe within one year. This study adds compelling evidence to the argument for policies that improve access to care and provide adequate financial protection from the costs of illness.

**Electronic supplementary material:**

The online version of this article (doi:10.1186/s12916-015-0433-1) contains supplementary material, which is available to authorized users.

## Background

The Association of Southeast Asian Nations (ASEAN) region consists of ten countries – Brunei, Cambodia, Indonesia, Laos, Malaysia, Myanmar, the Philippines, Singapore, Thailand, and Vietnam – and is home to over half a billion people. The burden of cancer is increasing in the ASEAN region, due to population ageing and growth and the adoption of cancer-associated lifestyle behaviours [1]. In 2012, there were estimated to be over 750,000 new cases of cancer, and incidence is expected to rise to 1.3 million per year by 2030 [2]. Survival rates for most cancers are poor and quality of life is greatly impaired [2–4]. In addition to this significant disease burden, cancer can have a profound economic effect on individuals and their households, especially among the poor and under-insured [5].

Most studies examining the economic burden of cancer have, however, been conducted in high-income settings. Little is known about its economic impact in low- and middle-income settings, where the financial implication of a cancer diagnosis may not be equitable because out-of-pocket (OOP) payments are the principal means of financing health care [6]. This not only relates to primary treatment, but may include long-term costs of adjuvant therapy and follow-up care [7–9]. Hence, a cancer diagnosis can quickly result in catastrophic payments for a household; that is, spending a disproportionate amount of household income on cancer treatment [10]. Furthermore, patients may be unable to continue working due to the burden of their symptoms, treatment, or side-effects, leading to poorer economic circumstances [11].

Health insurance is seen as an important means in offering households protection from catastrophic payments for illness. However, the extent of financial protection through insurance depends on which health services are covered and the level of subsidy offered. In the ASEAN region, while population coverage varies between 8 % (Laos) and 100 % (Malaysia), all countries – including those with universal health coverage – rely heavily on OOP financing [12, 13].

Despite the risk of a cancer epidemic overwhelming the region, governments have been slow to react to the health consequences of socioeconomic and demographic changes. Hence, in 2011, two regional initiatives were launched to increase cancer awareness and inform priority setting. First, a series of roundtable meetings of key stakeholders and experts were organised to generate knowledge and interest through engagement with the media [14, 15]. Second, a study of new cancer patients in eight countries in the ASEAN region (Cambodia, Indonesia, Laos, Malaysia, Myanmar, the Philippines, Thailand, and Vietnam) was instigated – the Asean CosTs In ONcology (ACTION) study – to assess the economic and health impact of cancer. This paper presents the main results.

## Methods

### Ethical approval

The ACTION study was approved by the University of Sydney’s Human Research Ethics Committee. Approvals from local institutional ethics committees and other regional or national regulatory bodies were obtained prior to the initiation of the study in all centres (Additional file 1). Written informed consent, complying with local, regional, and national requirements, was obtained from all participants prior to entry into the study.

### Study design

ACTION was a prospective longitudinal study; detailed methods have been published previously [16]. In brief, patients diagnosed with a first time cancer were consecutively recruited (within 12 weeks from initial date of diagnosis) from 47 sites, including public and private hospitals and cancer centres. Patients were aged 18 years and older, aware of their cancer diagnosis, and willing to participate in follow-up interviews. Participants were interviewed (face-to-face or by telephone) at baseline, 3, and 12 months after diagnosis. Questionnaires were translated into local languages.

### Baseline measures and key outcomes

Data were collected on age, sex, marital status, country of residence, highest level of education attained, employment status, recent experience of economic hardship (whether in the previous 12 months they were unable to make any necessary household payments (for example, food, housing) or needed assistance to do so) [17], annual household income, and health insurance status. Clinical characteristics, cancer site, and cancer stage (TNM classification) were obtained from medical records. Health-related quality of life was assessed using the EuroQol (EQ-5D) [18]. Further details are given in the study protocol [16].

The primary outcome at 12 months was financial catastrophe (FC) following treatment for cancer, defined as OOP costs at 12 months equal to or exceeding 30 % of annual household income [19, 20]. OOP costs represented hospital and non-hospital health care costs which were directly incurred by patients at point of delivery and not reimbursed by insurance. Participants prospectively completed a cost diary for the duration of the study. The second key outcome was all-cause mortality. FC and death were recorded at both follow-up interviews.

### Statistical analyses

Multinomial regression models were used to estimate odds ratios (ORs) and 95 % confidence intervals (CIs) for death and FC, relative to being alive without experiencing FC, thus allowing for death as a competing risk to FC. Baseline characteristics considered for association with these joint outcomes were socio-demographic (age, sex, and level of education), economic (household income grouped into low (0–75 % of mean national income), middle (75–125 %), and high income (>125 %), insurance status (yes or no), experience of economic hardship, and paid work status), and clinical (baseline health-related quality of life, cancer site – separately by sex – and cancer stage) [21]. Due to small numbers for some cancer sites, sites were grouped into body location or system: digestive/gastrointestinal; breast; gynaecological; head and neck; haematological/blood; respiratory/thoracic; and other cancers. Analyses were adjusted for age, sex, cancer stage, and geographic region, grouped as low (Cambodia, Myanmar), low-middle (Indonesia, Laos, Vietnam, the Philippines), and upper-middle income (Thailand, Malaysia). Participants who experienced FC at 3 months, but could not be contacted at 12 months, were coded as having experienced FC at 12 months. Primary analyses were conducted on participants with complete data on outcome status at 12 months. More extreme cut-offs for household income groups were tested in a sensitivity analysis: low (0–50 % of mean national income); middle (50–150 %); and high income (>150 %). Furthermore, multiple imputation (m = 5) using predictive mean matching was carried out to impute the missing data on the outcome variables. The imputation models included the outcome variables themselves, all socio-demographic, clinical, and economic predictors examined, and country [22]. Analyses were performed using STATA, version 12.0 (Stata, College Station, TX, USA), and R, version 2.15.3 (R Foundation for Statistical Computing, Vienna, Austria).

## Results

Between March 2012 and September 2013, after exclusions due to patient or doctor refusals, 9,513 patients were recruited into the study. The mean age was 52 years, 64 % were women, 61 % had attained at least secondary education, and 45 % had some form of health insurance. The most common cancer site recorded was breast (26 %); the greatest number was recruited in Indonesia (Table 1). For patients with available data on cancer stage (n = 5,159), 11 % presented with stage I, 31 % with stage II, 33 % with stage III, and 24 % with stage IV cancers. Haematological cancers were diagnosed in 825 patients (Additional file 2: Table S1).

**Table 1:** Demographic, socioeconomic, and clinical characteristics of the study population (n = 9,513)

Characteristic	n	%
Age (years)		
<45	2,780	29
45–54	2,801	30
55–64	2,463	26
≥65	1,467	15
Missing	2	<1
Sex		
Male	3,470	37
Female	6,043	63
Marital status		
Married	7,352	77
Not married	2,154	23
Missing	7	<1
Level of education		
0–6 years (primary)	3,693	39
7–12 years (secondary)	3,817	40
>12 years (tertiary)	1,992	21
Missing	11	<1
Country of residence		
Cambodia	206	2
Indonesia	2,335	25
Laos	101	1
Malaysia	1,662	18
Myanmar	1,178	12
Philippines	909	10
Thailand	1,206	13
Vietnam	1,916	20
Household size		
1–2	1,337	14
3–5	5,555	58
>5	2,570	27
Missing	9	<1
Household income (of mean national income)		
0–25 %	1,103	12
25–50 %	1,185	13
50–75 %	1,031	11
75–100 %	1,020	11
100–125 %	767	8
125–150 %	381	4
150–175 %	427	5
175–200 %	417	4
>200 %	1,819	19
Not known	1,336	14
Missing	27	<1
Main source of household income		
Crops and agricultural sidelines	1,965	21
Family business	1,287	14
Wages	4,568	48
Remittances and gifts	455	5
Other income	1,213	13
Missing	25	<1
Health insurance status		
Government-provided insurance	3,061	32
Employment-based insurance	568	6
Private insurance	857	9
Other community insurance	65	<1
None	5,237	55
Missing	11	<1
Type of hospital		
Public	8,760	92
Private	610	6
Other (for example, military)	143	2
Experienced economic hardship in the year before diagnosis		
Yes	5,146	54
No	4,352	46
Missing	15	<1
Paid work (patient level) before diagnosis (self-employed or for a wage)		
Yes	4,512	47
No	4,992	53
Missing	9	<1
Cancer site		
Mouth and pharynx	1,063	11
Oesophagus	160	2
Stomach	305	3
Colon and rectum	910	10
Liver	83	1
Pancreas	53	<1
Trachea, bronchus, and lung	623	7
Melanoma	40	<1
Female breast	2,445	26
Cervix	1,005	11
Uterus	177	2
Ovary	242	3
Prostate	47	<1
Bladder	60	<1
Lymphomas and multiple myeloma	454	5
Leukaemia	371	4
Other malignant neoplasms	1,295	14
Missing	179	2
Cancer stage		
Stage I	590	6
Stage II	1,613	17
Stage III	1,696	18
Stage IV	1,260	13
None (haematological cancers)	825	9
Missing	3,529	37

**Fig. 1:**
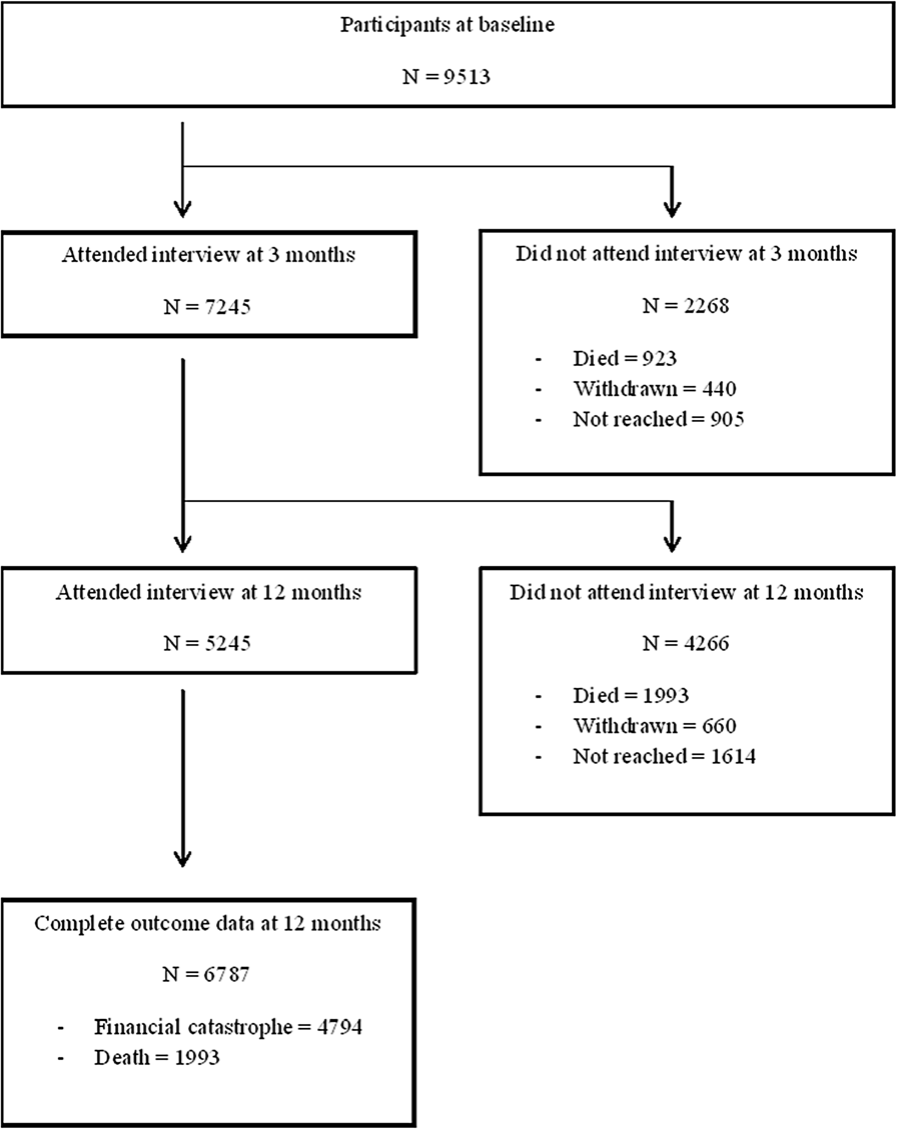
Participant flowchart

The follow-up interviews at 3 and 12 months were completed by 7,245 (76 %) and 5,245 (55 %) participants, respectively. At 12 months, 1,993 (29 %) participants had died. Complete outcome data (data on FC and death) were available for 6,787 participants (71 %) (Fig. 1).

Participants with incomplete outcome data (n = 2,726) were slightly younger (51 versus 52 years), more likely to be male (38 versus 33 %), and less likely to have a high income (17 versus 38 %), compared to those with complete outcome data (all *P* values <0.001). There were no significant differences in other socio-demographic, clinical, or economic characteristics.

**Fig. 2:**
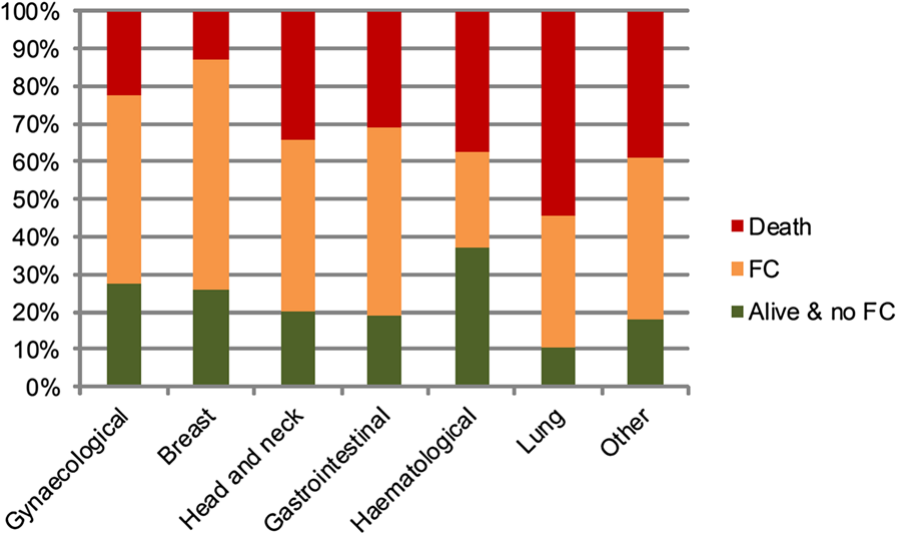
Competing outcomes of death, financial catastrophe, and alive with no financial catastrophe at 12 months after diagnosis, by location of cancer in the body

At 12 months, 3,248 participants (48 % of those with complete outcome data) experienced FC and 1,546 (23 %) were alive and did not experience FC. Survival without FC was most frequent in participants with haematological cancer (37 %), gynaecological cancer (27 %), and breast cancer (26 %) (Fig. 2).

After controlling for confounding variables, women had lower odds of death (OR, 0.62; 95 % CI, 0.51–0.75) than men, but sex was not significantly associated with FC, relative to the reference outcome (alive and no FC) (Table 2). Age of >65 years was associated with a higher odds of FC (1.51; 1.17–1.94) and death (2.64; 2.00–3.49), compared to age <45 years. Being unmarried was also associated with a higher odds of FC (1.09; 1.09–1.60) and death (1.42; 1.15–1.77), compared to participants who were married. Having completed primary education only, compared to tertiary education, was significantly associated with a higher odds of FC (1.45; 1.16–1.82) and death (2.50; 1.93–3.25).

**Table 2:** Odds ratios (and 95 % confidence intervals) for financial catastrophe and death, relative to no financial catastrophe (reference) in all participants with complete outcome data (n = 6,787), adjusted for age, sex, cancer stage, and geographic region

Characteristic		Financial catastrophe	Death
Age (years)	<45	Reference	Reference
	45–54	1.05 (0.85–1.30)	1.08 (0.84–1.38)
	55–64	1.41 (1.13–1.75)	1.59 (1.23–2.04)
	≥65	1.51 (1.17–1.94)	2.64 (2.00–3.49)
Sex	Men	Reference	Reference
	Women	1.14 (0.96–1.36)	0.62 (0.51–0.75)
Highest level of education	Tertiary	Reference	Reference
	Secondary	1.44 (1.16–1.79)	1.43 (1.11–1.85)
	Primary	1.45 (1.16–1.82)	2.50 (1.93–3.25)
Marital status	Married	Reference	Reference
	Unmarried	1.32 (1.09–1.60)	1.42 (1.15–1.77)
Health insurance	Yes	Reference	Reference
	No	1.27 (1.05–1.52)	1.51 (1.21–1.88)
Economic hardship	No	Reference	Reference
	Yes	1.40 (1.19–1.64)	1.82 (1.51–2.20)
Income level	High	Reference	Reference
	Middle	2.15 (1.73–2.67)	1.91 (1.47–2.47)
	Low	5.86 (4.76–7.23)	5.52 (4.34–7.02)
Paid work	Yes	Reference	Reference
	No	1.32 (1.11–1.56)	1.60 (1.31–1.94)
Cancer region: females	Digestive/gastrointestinal	Reference	Reference
	Breast	0.99 (0.69–1.41)	0.45 (0.29–0.69)
	Gynaecological	0.73 (0.49–1.08)	0.69 (0.43–1.11)
	Head and neck	0.69 (0.40–1.19)	0.65 (0.35–1.18)
	Haematological/blood	0.90 (0.69–1.19)	1.93 (1.39–2.69)
	Respiratory/thoracic	1.36 (0.65–2.85)	2.28 (1.07–4.86)
	Other	0.83 (0.48–1.41)	0.99 (0.54–1.83)
Cancer region: males	Digestive/gastrointestinal	Reference	Reference
	Head and neck	0.54 (0.36–0.80)	0.36 (0.24–0.55)
	Haematological/blood	0.56 (0.42–0.76)	1.10 (0.80–1.51)
	Respiratory/thoracic	1.18 (0.67–2.09)	1.88 (1.07–3.31)
	Other	0.56 (0.37–0.86)	0.65 (0.41–1.01)
Cancer stage	I	Reference	Reference
	II	1.23 (0.94–1.60)	0.93 (0.65–1.34)
	III	1.23 (0.94–1.62)	2.34 (1.65–3.33)
	IV	1.52 (1.12–2.05)	5.43 (3.77–7.82)
	None (haematological cancers)	0.68 (0.49–0.95)	3.04 (2.07–4.46)
EQ-5D score (per 0.1 decrement)		1.11 (1.07–1.16)	1.24 (1.18–1.30)

Participants in the low income category within each country had significantly higher odds of FC (5.86; 4.76–7.23) and death (5.52; 4.34–7.02) than participants with high income, relative to being alive and no FC. Using more extreme cut-offs for low and high household income (0–50 % of the mean national income for a low income and >150 % for a high income) resulted in higher odds of FC (9.16; 7.07–11.87) and death (9.30; 6.95–12.44) for the low income category. The country-region specific analysis showed that a low income is especially a factor in predicting FC in the upper-middle income countries (13.75; 10.21–18.51) and less so in lower-middle income countries (1.97; 1.38–2.82) (Additional file 2: Table S2a and S2b). Not having paid work also increased the odds of FC (1.32; 1.11–1.56) and death (1.60; 1.31–1.94). Having some form of health insurance provided protection from FC; those without insurance were more likely to experience FC than those with insurance (1.27; 1.05–1.52). Participants without health insurance were more likely to die (1.51; 1.21–1.88), relative to being alive and not experiencing FC; health insurance was inversely related to FC in upper-middle income countries only.

Cancer stage IV at diagnosis was significantly associated with a higher odds of FC (1.52; 1.12–2.05) and death (5.43; 3.76–7.82), compared to stage I. In terms of health-related quality of life, a decrement of 0.1 point as assessed on the EQ-5D was associated with higher odds of FC (1.11; 1.07–1.16) and death (1.24; 1.18–1.30).

In females, cancer site was not associated with FC. In males, cancer in the head and neck region (0.54; 0.36–0.80) and haematological cancers (0.56; 0.42–0.76) were associated with a lower odds of FC compared to digestive cancers (reference group).

Sensitivity analyses employing missing value imputation (Additional file 2: Table S3) did not change the inferences, except that the effect of health insurance on the odds of FC became non-significant at the conventional 5 % level.

## Discussion

To our knowledge, the ACTION study is the largest observational study of the household burden of cancer yet conducted in Asia. A year after diagnosis, almost a third of patients affected by cancer in the ASEAN region died and almost a half of their households faced catastrophic health care expenses. Patients with advanced stages of cancer at diagnosis and socioeconomically disadvantaged cancer patients, including those with primary education only, low income, and no health insurance, were more likely to experience FC or die within 12 months.

This research adds compelling evidence to the argument for effective cancer control policies and timely access to affordable treatment in low- and middle-income countries. Previously, evidence of significant household economic burden due to cancer has come from only a few, small cross-sectional studies [23, 24]. There has, however, been increasing attention given to the economic impact of non-communicable diseases in low- and middle-income settings, with two recent reviews highlighting the heavy financial burden that such diseases pose on affected households [25, 26]. In a review of studies that reported on expenditures on chronic diseases, mean expenditures ranged from 5 % to 59 % of household income, household total health expenditure, and household non-food expenditure, but results on catastrophic health expenditures were not reported [26]. A literature review on the costs imposed by non-communicable diseases in low- and middle-income settings included 19 studies that reported on OOP health expenditure as a percentage of capacity to pay or total household expenditure due to health shocks, and found that between 0 % and 34 % of the study population experienced FC, depending on the methods used [25]. Comparison of these findings with our results is difficult due to differences in defining catastrophic spending: some studies used a threshold OOP share of total household expenditure; others of household ‘capacity to pay’; or of ‘non-food expenditure’. In addition, the threshold used also varies, ranging from 10 % to 40 %. Furthermore, in the majority of the above-mentioned studies, OOP estimates were based on retrospective recall of health care utilisation in household surveys, while our study used a prospective cost diary. Studies have shown that OOP estimates depend heavily on the measures used and length of recall periods [27, 28]. Compared to prospective cost diaries, health care utilisation is generally under-reported in household surveys [27]. Nonetheless, results from this study, taken together with other studies, signal the potential for cancer to result in a significant economic burden.

Women were less likely to die in the year following a cancer diagnosis than men, but no significant association between the patient’s sex and their household’s odds of experiencing FC was found. Better survival rates for female cancers may be explained by the high proportion of breast cancer in this population, and its relatively good prognosis, while colorectal, mouth and lung cancers, with a generally poor prognosis [29], were most common in men. The risk of FC increases with age, perhaps due to increasing co-morbidities which result in greater complexity of the illness and treatment. As expected, age was significantly associated with the risk of death at 12 months. A more advanced cancer stage at diagnosis was associated with higher odds of FC and death.

We found that having a below average income, no health insurance, not having paid work, having experienced economic hardship prior to diagnosis, and having experienced no more than primary education, were all associated with a higher odds of experiencing FC. Household income showed the strongest association, with these patients having more than five times the odds of FC when an income <75 % of the mean national income was considered a low income, and even nine times the odds when an income <50 % was used as the threshold. That this gradient was found to be more pronounced in upper-middle compared to lower-middle income countries suggests that the risk of FC posed by having a low income is as much based on relative as opposed to absolute disadvantage.

The relationship between health insurance and FC found in the primary analyses of this study was not particularly strong, and was non-significant in the sensitivity analysis where missing data were imputed. Analyses by level of economic development provided some explanation to these inconclusive results: in upper-middle income countries (Malaysia and Thailand) health insurance did provide significant protection from FC; but in lower-middle income countries it did not. This may be explained by the limitations of benefit packages available through health insurance programs in some of the participating lower-middle income countries, which has been well-recognised as a problem in Vietnam and the Philippines [13]. Since health insurance status was assessed as a categorical variable it was not possible to take into account variations in level of coverage.

The findings in relation to the socioeconomic variables reinforce the well-founded conclusions that can be drawn from the social determinants literature – those at greater levels of disadvantage tend to have higher risks of financial hardship and poor health [30]. Reflecting this was the strong relationship of various socioeconomic indicators and death within 12 months. This, and the observed association between low quality of life and higher odds of FC, underscores the relationship between underlying economic disadvantage, health, and economic outcomes in cancer.

The study has a number of limitations. We did not recruit a random cross-section of people with incident cancer in the region due to a variety of reasons. First, as we could only identify cases once individuals presented to hospital, we potentially excluded individuals who did not seek hospital treatment due to geographical isolation, poverty, or socio-cultural barriers [31]. Second, clinicians responsible for enrolling patients into the study appear to have under-recruited those with the most virulent types of cancer, such as lung and liver cancers. Third, public awareness of some types of cancer, specifically breast cancer, was greater than for others, which is likely to have additionally motivated certain cancer patients, particularly women, to agree to participate in the study. Furthermore, patients treated in private hospitals were under-represented in the study (6 %) and it is unclear whether this has introduced a bias in our estimates of the level of FC. Although private hospitals have often been observed to generate the highest OOP expenses [25] they also tend to attract patients with a higher income. All these factors compromise the generalisability of some of our results, and probably means that we have underestimated the 12-month rate of death from all cancers, but is unlikely to invalidate the main conclusions. Another drawback is that 2,767 participants (29 %) lacked at least one component of data on death, household income, or OOP costs required to compute the study outcomes. The challenges of eliciting income and other socioeconomic data have been well-documented [32], and incomplete follow-up due to being unable to contact many subjects in rural areas, despite repeated telephone calls and field visits, is inevitable in the region studied. The findings from the sensitivity analysis, in which multiple imputation was used to impute the missing data, did not vary substantially from the non-imputed findings, and would not alter conclusions.

These drawbacks have to be considered in the light of the paucity of cancer statistics from the region sampled [2, 15, 33]. The study benefited from having a large sample of patients with various cancer sites and cancer stages from eight countries which have disparate health systems. Due to the large size of the study, it was possible to produce reliable estimates of the influence of a range of demographic, socioeconomic, and clinical predictors. In addition, the study’s longitudinal approach improved on most previous economic studies which used cross-sectional surveys based on retrospective reporting of costs, as well as much smaller sample sizes, with subsequent jeopardy for both bias and sampling error. Furthermore, using a multinomial logistic regression model, we were able to adjust FC for the competing outcome of death. This is important as studies that have previously examined the burden to households associated with illnesses have generally focused exclusively on ‘economic’ outcomes in terms of OOP costs and FC [10, 25, 26], but have overlooked a crucial reason why patients may avoid, or not report incurring, high OOP costs, that is, they may die, and this is unlikely to be non-informative censoring.

## Conclusions

This study provides the type of precise evidence that is required to develop effective policies and programs to address the overall burden of cancer care in the ASEAN region, with potential generalisation elsewhere in the developing world. The results show that a cancer diagnosis is disastrous, even within only 12 months, for over 75 % of new patients. Socioeconomically disadvantaged cancer patients and patients with advanced cancer stages at diagnosis were common and particularly vulnerable to adverse economic outcomes and poor survival. The need for more resources to aid early detection as well as policies that improve access to care, by removing financial barriers and providing adequate financial protection from the costs of illness, is clear.

## Key message

Over 75 % of new cancer patients in Southeast Asia experience financial catastrophe or die within one year. An advanced stage at diagnosis and socioeconomic disadvantage are significant risk factors for these poor outcomes. There is an urgent need for more resources to aid early detection and policies aimed to provide adequate financial protection from the costs of cancer.

## Appendix

*Writing committee*

Merel Kimman^1,2,*^

Email: mkimman@georgeinstitute.org.au

Stephen Jan^1^

Cheng Har Yip^3^

Hasbullah Thabrany^4^

Sanne A Peters^5^

Nirmala Bhoo-Pathy^6,7^

Mark Woodward^1,5,**^

Phone: +61 2 99934514

Email: markw@georgeinstitute.org.au

^1^The George Institute for Global Health, University of Sydney, Level 10, King George V Building, 83–117 Missenden Road, Camperdown, NSW 2050, Australia

^2^Department of Clinical Epidemiology and Medical Technology Assessment, Maastricht University Medical Centre, Maastricht, the Netherlands

^3^Department of Surgery, University of Malaysia Medical Centre, Kuala Lumpur, Malaysia

^4^Center for Health Economics and Policy Studies, Universitas Indonesia, Jakarta, Indonesia

^5^The George Institute for Global Health, Nuffield Department of Population Health, University of Oxford, Oxford, UK

^6^National Clinical Research Centre, Ministry of Health, Kuala Lumpur, Malaysia

^7^Faculty of Medicine, University of Malaya, Kuala Lumpur, Malaysia

^*^Corresponding author. Department of Clinical Epidemiology and Medical Technology Assessment, Maastricht University Medical Centre, Maastricht, the Netherlands

^**^Corresponding author. The George Institute for Global Health, Nuffield Department of Population Health, University of Oxford, Oxford, UK

*Principal investigators*

Phetsamone Arounlangsy (Lao Cancer Center, Department of Health Care, Ministry of Health, Laos), Soe Aung (Oncology Society, Myanmar Medical Association, Yangon, Myanmar), Soledad L Balete (Section of Medical Oncology, Jose R Reyes Memorial Medical Centre, Manila, Philippines), Nirmala Bhoo-Pathy (National Clinical Research Centre, Ministry of Health, Malaysia; Faculty of Medicine, University of Malaya, Kuala Lumpur, Malaysia), Bounthaphany Bounxouei (Mahosot Hospital, Laos), Dieu Bui (K Hospital, Vietnam), Jay Datukan (Section of Medical Oncology, St Luke’s Medical Centre, Quezon City, Metro Manila, Philippines), Agnes E Gorospe, (Section of Medical Oncology, St Luke’s Medical Centre, Quezon City, Metro Manila, Philippines), Cheng Har Yip (University of Malaya, Kuala Lumpur, Malaysia), Prasit Khopaibul (Suratthani Cancer Centre, Suratthani, Thailand), Thanut Khuayjarernpanishk (Ubonratchathani Cancer Centre, Ubonratchathani, Thailand), Thiravud Khuhaprema (National Cancer Institute of Thailand, Bangkok, Thailand), Myo Khin (Department of Medical Research, Lower Myanmar, Myanmar), David Kingston, Tawin Klinwimol (Ubonratchathani Cancer Centre, Ubonratchathani, Thailand), Somkiet Lalitwongsa (Lampang Cancer Hospital, Lampang, Thailand), Dhanoo Lawbundis (Lopburi Cancer Hospital, Lopburi, Thailand), Conrado Lola (Section of Medical Oncology, National Kidney and Transplant Institute, Quezon City, Metro Manila, Philippines), Leo Marbella (Section of Medical Oncology, National Kidney and Transplant Institute, Quezon City, Metro Manila, Philippines), Khoa Mai Trong (Bach Mai Hospital, Vietnam), Soe Oo Maung (Department of Radiotherapy, Yangon General Hospital, Yangon, Myanmar), Shu Mon (Bahosi Hospital, Myanmar), Win Pa Pa Naing (Department of Medical Research, Lower Myanmar, Myanmar), Corazon A Ngelangel (Section of Medical Oncology, University of the Philippines – College of Medicine, Philippine General Hospital, Manila, Philippines), Htun Lwin Nyein (Haematology Department, Yangon General Hospital, Yangon, Myanmar), Annielyn Beryl Ong-Cornel (Veterans Memorial Medical Centre, Quezon City, Philippines), Khin May Oo (Department of Medical Research, Lower Myanmar, Myanmar), Irisyl Orolfo-Real (Section of Medical Oncology, University of the Philippines – College of Medicine, Philippine General Hospital, Manila, Philippines), Dung Pham Xuan (Oncology Hospital, Ho Chi Minh city, Vietnam), Seang Pharin (Department of Onco-Hematology, Calmette Hospital, Cambodia), Pujianto (Department of Health Policy and Administration, School of Public Health, Universitas Indonesia, Indonesia), Oudayvone Rattanavong (Mahosot Hospital, Laos), Kouy Samnang (Department of Oncology, Khmer-Soviet Friendship Hospital, Cambodia), Somphob Sangkittipaiboon (Lopburi Cancer Hospital, Lopburi, Thailand), Suleeporn Sangrajrang, (National Cancer Institute of Thailand, Bangkok, Thailand), Cherelina Santiago-Ferreras (Section of Medical Oncology, Veterans Memorial Medical Centre, Quezon City, Metro Manila, Philippines), San Shwe (Department of Medical Research, Lower Myanmar, Myanmar), Eav Sokha (National Cancer Centre, Calmette Hospital, Cambodia), Thanadej Sinthusake (Mahavajiralongkorn Thanyaburi Hospital, Pathumthani, Thailand), Darunee Suanplu (Suratthani Cancer Hospital, Suratthani, Thailand), Jitraporn Tanabodee (Chonburi Cancer Hospital, Chonburi, Thailand), Hasbullah Thabrany (Center for Health Economics and Policy Studies, Universitas Indonesia, Indonesia), Kitisak Thepsuwan (Chonburi Cancer Hospital, Chonburi, Thailand), Yin Yin Tun (Department of Medicine, No 2 Military Hospital, Myanmar), Heng Viroath (Department of Oncology, Khmer-Soviet Friendship Hospital, Cambodia), Le Le Win (Department of Medical Research, Lower Myanmar), Swe Swe Win (Department of Oral Medicine, University of Dental Medicine, Yangon, Myanmar), and Tin Moe Win (Mandalay General Hospital, Myanmar).

*Other contributors*

Ami Ashariati (Soetomo Hospital, Indonesia), Djumhana Atmakusuma (Cipto Mangunkusumo Hospital, Indonesia), I Made Bakta (Sanglah Hospital, Indonesia), Loan Dang Thi Kim (Oncology Hospital Ho Chi Minh city, Vietnam), Phung Dang Thi Ngoc (Oncology Hospital Ho Chi Minh city, Vietnam), Tuan Diep Bao (Oncology Hospital Ho Chi Minh city, Vietnam), Ario Djatmiko (Surabaya Oncology Hospital, Indonesia), Andi Fachruddin (Wahidin Hospital, Indonesia), Johan Kurnianda (Sardjito Hospital, Indonesia), Helen Monaghan (The George Institute for Global Health, Australia), Abdul Muthalib (Medistra Hospital, Indonesia), Trang Ngo Thuy (Bach Mai Hospital, Vietnam), Thao Nguyen Hoang, (K Hospital, Vietnam), Nga Nguyen Thi Hoai (K Hospital, Vietnam), Sonar S Panigoro (Dharmais Cancer Center Hospital, Indonesia), Huy Phạm Quang (K Hospital, Vietnam), Goh Pik Pin (National Clinical Research Centre, Malaysia), Khanh Quach Thanh (Oncology Hospital Ho Chi Minh city, Vietnam), Prih Sarnianto (Universitas Indonesia, Indonesia), Dradjat R Suardi (Hasan Sadikin Hospital, Indonesia), Shridevi Subramaniam (National Clinical Research Centre, Malaysia), Aru W Sudoyo (MRCCC Siloam Hospital, Indonesia), Khoa Tran Dang (Ha Noi Oncology Hospital, Vietnam), Ha Tran Dinh (Bach Mai Hospital, Vietnam), Catharina Suharti (Karyadi Hospital, Indonesia), and Suyatno (Adam Malik Hospital, Indonesia).

*Institutions*

CAMBODIA: Calmette Hospital (Phnom Penh) and Khmer-Soviet Friendship Hospital (Phnom Penh). INDONESIA: Adam Malik Hospital (Medan), Cipto Mangunkusumo Hospital (Jakarta), Dharmais Cancer Center Hospital (Jakarta), Hasan Sadikin Hospital (Bandung), Karyadi Hospital (Semarang), Sanglah Hospital (Denpasar), Sardjito Hospital (Yogyakarta), Soetomo Hospital (Surabaya), Wahidin Sudirohusodo Hospital (Makassar), and Surabaya Oncology Hospital (Surabaya). LAOS: Mahosot Hospital (Vientiane). MALAYSIA: Hospital Ampang (Ampang), Hospital Kuala Lumpur (Kuala Lumpur), Hospital Melaka (Melaka), Hospital Queen Elizabeth I (Kota Kinabalu), Hospital Raja Perempuan Zainab II (Kota Baru), Hospital Seri Manjung (Seri Manjung), Hospital Sibu (Sibu), Hospital Sultanah Aminah (Johor Bahru), Hospital Sungai Buloh (Sungai Buloh), Hospital Tengku Ampuan Rahimah (Klang), Hospital Tuanku Fauziah (Kangar), Hospital Wanita dan Kanak-Kanak Sabah (Likas), Sime Darby Medical Centre (Subang Jaya), National Clinical Research Centre (Kuala Lumpur), University Malaya Medical Centre (Kuala Lumpur), and University Malaya Specialist Centre (Kuala Lumpur). MYANMAR: Bahosi Hospital (Yangon), Mandalay General Hospital (Mandalay), No 2 Military Hospital (Yangon), University of Dental Medicine (Yangon), and Yangon General Hospital (Yangon). PHILIPPINES: Jose R Reyes Memorial Medical Centre (Manila), University of the Philippines – College of Medicine Philippine General Hospital (Manila), National Kidney and Transplant Institute (Quezon City), St Luke’s Medical Centre (Quezon City), and Veterans Memorial Medical Centre (Quezon City). THAILAND: Chonburi Cancer Hospital (Chonburi), Lampang Cancer Hospital (Lampang), National Cancer Institute of Thailand (Bangkok), Lopburi Cancer Hospital (Lopburi), Mahavajiralongkorn Thanyaburi Hospital (Pathumthani), Suratthani Cancer Centre (Suratthani), and Ubonratchathani Cancer Hospital (Ubonratchathani) VIETNAM: Bach Mai Hospital (Hanoi), K Hospital (Hanoi), and Oncology Hospital Ho Chi Minh city.

*Executive committee*

Nirmala Bhoo-Pathy, Bounthaphany Bounxouei, Gloria Cristal-Luna, Nguyen Chan Hung, Myo Khin, Thiravud Khuhaprema, Merel Kimman, David Kingston, Stephen Jan, Eav Sokha, Hasbullah Thabrany, and Mark Woodward.

*Secretariat*

Helen Monaghan, Merel Kimman, Stephen Jan, and Mark Woodward.

## Additional files

Additional file 1:
**Overview approvals local ethics boards ACTION Study.** (DOCX 14 kb) Additional file 2: Table S1. Cancer stage by cancer site (n = 5,984). Cancer stage was not available for 3,529 participants. Table S2a. Odds ratios (and 95 % confidence intervals) for financial catastrophe and death, relative to no financial catastrophe (reference) in participants from lower-middle income countries, adjusted for age, sex, cancer stage, and geographic region. Table S2b. Odds ratios (and 95 % confidence intervals) for financial catastrophe and death, relative to no financial catastrophe (reference) in participants from upper-middle income countries, adjusted for age, sex, cancer stage, and geographic region. Table S3. Odds ratios (and 95 % confidence intervals) for financial catastrophe and death, relative to no financial catastrophe (reference) in all participants (n = 9,513), using missing value imputation and adjusted for age, sex, cancer stage, and geographic region. (DOCX 36 kb)

## Abbreviations

ACTION: Asean CosTs In Oncology
ASEAN: Association of Southeast Asian Nations
CI: Confidence interval
FC: Financial catastrophe
OOP: Out-of-pocket
OR: Odds ratio
