# Supplementary material for: Catastrophic health expenditure and 12-month mortality associated with cancer in Southeast Asia: results from a longitudinal study in eight countries
Source: BMC Med. 2015 Aug 18;13:190. doi: 10.1186/s12916-015-0433-1 (PMC4539728; doi:10.1186/s12916-015-0433-1)
Supplement: Additional file 1: — Overview approvals local ethics boards ACTION Study. (DOCX 14 kb) [file 12916_2015_433_MOESM1_ESM.docx]

**Additional file 1**

**Overview approvals local ethics boards ACTION Study**

**Cambodia:**

National Ethics Committee for Health Research - Ministry of Health

**Indonesia:**

The Committee on Health Research Ethics - National Institute of Health Research and

Development - lndonesia Ministry of Health

**Laos:**

National Ethics Committee for Health Research – Council of Medical Sciences – Ministry of Health - Lao People’s Democratic Republic

**Malaysia:**

Office of the Deputy Director-General of Health – Research and Technical Support - Ministry of Health Malaysia

**Myanmar:**

Ethical Review Committee – Department of Medical Research (Lower Myanmar) – Ministry of Health – The Government of The Republic of the Union of Myanmar

**Philippines:**

St. Luke’s Institutional Ethics Review Committee

UP Manila Research Ethics Board – University of the Philippines Manila

Institutional Ethics Review Committee – Jose R Reyes Memorial Medical Centre

National Kidney and Transplant Institute Technical Review Board Committee and Research Ethics Committee

Veterans Memorial Medical Center – Institutional Review Board

**Thailand:**

Ethics Committee of Lampang Cancer Hospital, Department of Medical Services, Ministry of Public Health

Ethics Committee of MahaVajiralongkorn Cancer Center

Ethics Committee of National Cancer Institute

Ethics Committee of Chonburi Cancer Hospital

Ethics Committee of Ubonratchathani Cancer Center

Lopburi Cancer Center Ethics Committee for Human Research

**Vietnam:**

Committee for Evaluation of Ethical Aspects in Biomedical Research - Ministry of Health
