# Supplementary material for: Catastrophic health expenditure and 12-month mortality associated with cancer in Southeast Asia: results from a longitudinal study in eight countries
Source: BMC Med. 2015 Aug 18;13:190. doi: 10.1186/s12916-015-0433-1 (PMC4539728; doi:10.1186/s12916-015-0433-1)
Supplement: Additional file 2: Table S1. — Cancer stage by cancer site (n = 5,984). Cancer stage was not available for 3,529 participants. Table S2a. Odds ratios (and 95 % confidence intervals) for financial catastrophe and death, relative to no financial catastrophe (reference) in participants from lower-middle income countries, adjusted for age, sex, cancer stage, and geographic region. Table S2b. Odds ratios (and 95 % confidence intervals) for financial catastrophe and death, relative to no financial catastrophe (reference) in participants from upper-middle income countries, adjusted for age, sex, cancer stage, and geographic region. Table S3. Odds ratios (and 95 % confidence intervals) for financial catastrophe and death, relative to no financial catastrophe (reference) in all participants (n = 9,513), using missing value imputation and adjusted for age, sex, cancer stage, and geographic region. (DOCX 36 kb) [file 12916_2015_433_MOESM2_ESM.docx]

Additional file 2.

Table S1. Cancer stage by cancer site (n = 5,984). Cancer stage was not available for 3,529 participants.

| **Cancer site** |  | **Stage I** | | **Stage II** | | **Stage III** | | **Stage IV** | | **None-staged** | |
| --- | --- | --- | --- | --- | --- | --- | --- | --- | --- | --- | --- |
|  | n | n | % | n | % | n | % | n | % | n | % |
| Mouth and pharynx | 768 | 56 | 7 | 91 | 12 | 189 | 25 | 432 | 56 | - | - |
| Oesophagus | 77 | 4 | 5 | 20 | 26 | 37 | 48 | 16 | 21 | - | - |
| Stomach | 171 | 23 | 14 | 30 | 18 | 51 | 30 | 67 | 39 | - | - |
| Colon and rectum | 619 | 61 | 10 | 128 | 21 | 295 | 48 | 135 | 22 | - | - |
| Liver | 46 | 2 | 4 | 2 | 4 | 26 | 57 | 16 | 35 | - | - |
| Pancreas | 27 | 2 | 7 | 12 | 44 | 4 | 15 | 9 | 33 | - | - |
| Trachea, bronchus, lung | 431 | 44 | 10 | 50 | 12 | 118 | 27 | 219 | 51 | - | - |
| Melanoma | 19 | 1 | 5 | 7 | 37 | 4 | 21 | 7 | 37 | - | - |
| Female breast | 1,843 | 198 | 11 | 868 | 47 | 616 | 33 | 161 | 9 | - | - |
| Cervix | 408 | 78 | 19 | 141 | 35 | 132 | 32 | 57 | 14 | - | - |
| Uterus | 87 | 26 | 30 | 10 | 12 | 25 | 29 | 26 | 30 | - | - |
| Ovary | 96 | 16 | 17 | 34 | 35 | 28 | 29 | 18 | 19 | - | - |
| Prostate | 15 | 3 | 20 | 1 | 7 | 2 | 13 | 9 | 60 | - | - |
| Bladder | 43 | 3 | 7 | 14 | 33 | 8 | 19 | 18 | 42 | - | - |
| Lymphomas and multiple myeloma | 454 | - | - | - | - | - | - | - | - | 454 | 100 |
| Leukaemia | 371 | - | - | - | - | - | - | - | - | 371 | 100 |
| Other malignant neoplasms | 509 | 73 | 14 | 205 | 40 | 161 | 32 | 70 | 14 | - | - |
| **Total** | **5,984** | **590** | **10** | **1,613** | **27** | **1,696** | **28** | **1,260** | **21** | **825** | **14** |

Table S2a. Odds ratios (and 95% confidence intervals) for financial catastrophe and death, relative to no financial catastrophe (reference) in participants from lower-middle income countries, adjusted for age, sex, cancer stage, and geographic region.

| Characteristic |  | Financial catastrophe | Death |  |
| --- | --- | --- | --- | --- |
| Age (years) | <45 | Reference | Reference | |
|  | 45–54 | 1.04 (0.71–1.54) | 1.10 (0.72–1.71) | |
|  | 55–64 | 1.26 (0.82–1.92) | 1.34 (0.84–2.12) | |
|  | ≥65 | 1.28 (0.74–2.24) | 1.89 (1.05–3.38) | |
|  |  |  |  | |
| Sex | Men | Reference | Reference | |
|  | Women | 1.27 (0.90–1.79) | 0.61 (0.42–0.87) | |
|  |  |  |  | |
| Highest level of education | Tertiary | Reference | Reference | |
|  | Secondary | 1.43 (1.00–2.05) | 1.76 (1.17–2.64) | |
|  | Primary | 1.56 (1.01–2.41) | 3.33 (2.07–5.33) | |
|  |  |  |  | |
| Marital status | Married | Reference | Reference | |
|  | Unmarried | 2.07 (1.25–3.43) | 1.46 (0.84–2.53) | |
|  |  |  |  | |
| Health insurance | Yes | Reference | Reference | |
|  | No | 0.76 (0.55–1.06) | 1.24 (0.87–1.76) | |
|  |  |  |  | |
| Economic hardship | No | Reference | Reference | |
|  | Yes | 1.28 (0.93–1.75) | 1.59 (1.12–2.26) | |
|  |  |  |  | |
| Income level | High | Reference | Reference | |
|  | Middle | 1.62 (1.04–2.52) | 1.85 (1.10–3.10) | |
|  | Low | 1.97 (1.38–2.81) | 3.47 (2.32–5.20) | |
|  |  |  |  | |
| Paid Work | Yes | Reference | Reference | |
|  | No | 1.41 (1.02–1.94) | 1.62 (1.14–2.30) | |
|  |  |  |  | |
| Cancer region | Digestive/ gastrointestinal | Reference | Reference | |
|  | Breast | 0.70 (0.40–1.24) | 0.43 (0.23–0.81) | |
|  | Gynaecological | 1.90 (0.61–5.92) | 1.93 (0.58–6.42) | |
|  | Head and neck | 0.53 (0.28–0.97) | 0.33 (0.18–0.64) | |
|  | Haematological/blood | 0.70 (0.45–1.08) | 1.36 (0.83–2.23) | |
|  | Respiratory/thoracic | 0.98 (0.42–2.29) | 2.14 (0.93–4.95) | |
|  | Other | 0.55 (0.30–1.00) | 0.65 (0.34–1.22) | |
|  |  |  |  | |
| Cancer stage | I | Reference | Reference | |
|  | II | 1.08 (0.57–2.02) | 1.06 (0.48–2.33) | |
|  | III | 0.63 (0.34–1.18) | 1.80 (0.85–3.81) | |
|  | IV | 0.70 (0.34–1.41) | 4.95 (2.20–11.16) | |
|  | None (haematological cancers) | 0.63 (0.29–1.40) | 2.33 (0.95–5.73) | |
|  |  |  |  | |
| EQ5D score (per 0.1 decrement) |  | 1.14 (1.05–1.24) | 1.27 (1.16–1.39) | |

Table S2b. Odds ratios (and 95% confidence intervals) for financial catastrophe and death, relative to no financial catastrophe (reference) in participants from upper-middle income countries, adjusted for age, sex, cancer stage, and geographic region.

| Characteristic |  | Financial catastrophe | Death |  |
| --- | --- | --- | --- | --- |
| Age (years) | <45 | Reference | Reference | |
|  | 45–54 | 1.02 (0.76–1.36) | 1.18 (0.80–1.75) | |
|  | 55–64 | 1.68 (1.26–2.23) | 1.77 (1.21–2.60) | |
|  | ≥65 | 1.68 (1.23–2.30) | 3.13 (2.14–4.59) | |
|  |  |  |  | |
| Sex | Men | Reference | Reference | |
|  | Women | 0.97 (0.77–1.21) | 0.60 (0.46–0.79) | |
|  |  |  |  | |
| Highest level of education | Tertiary | Reference | Reference | |
|  | Secondary | 1.83 (1.35–2.50) | 1.13 (0.72–1.76) | |
|  | Primary | 1.49 (1.09–2.03) | 2.68 (1.75–4.10) | |
|  |  |  |  | |
| Marital status | Married | Reference | Reference | |
|  | Unmarried | 1.14 (0.91–1.44) | 1.53 (1.14–2.05) | |
|  |  |  |  | |
| Health insurance | Yes | Reference | Reference | |
|  | No | 1.81 (1.42–2.31) | 1.23 (0.91–1.67) | |
|  |  |  |  | |
| Economic hardship | No | Reference | Reference | |
|  | Yes | 1.58 (1.29–1.95) | 1.45 (1.11–1.90) | |
|  |  |  |  | |
| Income level | High | Reference | Reference | |
|  | Middle | 3.16 (2.31–4.33) | 1.35 (0.94–1.95) | |
|  | Low | 13.75 (10.21–18.51) | 3.18 (2.26–4.47) | |
|  |  |  |  | |
| Paid Work | Yes | Reference | Reference | |
|  | No | 1.41 (1.13–1.76) | 1.43 (1.08–1.91) | |
|  |  |  |  | |
| Cancer region | Digestive/ gastrointestinal | Reference | Reference | |
|  | Breast | 0.81 (0.57–1.15) | 0.19 (0.10–0.35) | |
|  | Gynaecological | 0.41 (0.27–0.62) | 0.66 (0.40–1.11) | |
|  | Head and neck | 1.03 (0.68–1.56) | 1.15 (0.71–1.85) | |
|  | Haematological/blood | 0.71 (0.56–0.91) | 1.97 (1.38–2.80) | |
|  | Respiratory/thoracic | 1.34 (0.76–2.35) | 2.20 (1.21–3.99) | |
|  | Other | 0.62 (0.39–0.97) | 1.05 (0.63–1.75) | |
|  |  |  |  | |
| Cancer stage | I | Reference | Reference | |
|  | II | 1.30 (0.93–1.82) | 1.54 (0.78–3.02) | |
|  | III | 1.60 (1.14–2.25) | 4.38 (2.34–8.19) | |
|  | IV | 1.74 (1.21–2.52) | 9.34 (5.00–17.44) | |
|  | None (haematological cancers) | 0.66 (0.44–1.01) | 5.35 (2.80–10.20) | |
|  |  |  |  | |
| EQ5D score (per 0.1 decrement) |  | 1.13 (1.08–1.19) | 1.16 (1.09–1.23) | |

Table S3. Odds ratios (and 95% confidence intervals) for financial catastrophe and death, relative to no financial catastrophe (reference) in all participants (n = 9,513), using missing value imputation and adjusted for age, sex, cancer stage, and geographic region.

| Characteristic |  | Financial catastrophe | Death |
| --- | --- | --- | --- |
| Age (years) | <45 | Reference | Reference |
|  | 45–54 | 1.08 (0.85–1.38) | 1.19 (0.94–1.51) |
|  | 54–64 | 1.29 (1.03–1.62) | 1.50 (1.17–1.93) |
|  | ≥65 | 1.30 (1.01–1.68) | 2.48 (1.90–3.25) |
|  |  |  |  |
| Sex | Men | Reference | Reference |
|  | Women | 1.18 (1.00–1.40) | 0.55 (0.45–0.68) |
|  |  |  |  |
| Highest level of education | Tertiary | Reference | Reference |
|  | Secondary | 1.34 (1.12–1.59) | 1.42 (1.13–1.77) |
|  | Primary | 1.34 (1.11–1.62) | 2.17 (1.69–2.77) |
|  |  |  |  |
| Marital status | Married | Reference | Reference |
|  | Unmarried | 1.24 (1.04–1.49) | 1.32 (1.07–1.62) |
|  |  |  |  |
| Health insurance | Yes | Reference | Reference |
|  | No | 1.10 (0.92–1.33) | 1.34 (1.10–1.64) |
|  |  |  |  |
| Economic hardship | No | Reference | Reference |
|  | Yes | 1.28 (1.11–1.47) | 1.58 (1.33–1.88) |
|  |  |  |  |
| Income level | High | Reference | Reference |
|  | Middle | 1.83 (1.50–2.25) | 1.66 (1.29–2.15) |
|  | Low | 4.09 (3.31–5.06) | 3.83 (2.93–5.00) |
|  |  |  |  |
| Paid Work | Yes | Reference | Reference |
|  | No | 1.26 (1.07–1.49) | 1.33 (1.12–1.58) |
|  |  |  |  |
| Cancer region females | Digestive/ gastrointestinal | Reference | Reference |
|  | Breast | 1.07 (0.78–1.48) | 0.45 (0.29–0.68) |
|  | Gynaecological | 0.77 (0.54–1.11) | 0.62 (0.40–0.98) |
|  | Head and neck | 0.92 (0.56–1.51) | 0.79 (0.45–1.39) |
|  | Haematological/blood | 1.30 (0.77–2.18) | 3.65 (1.94–6.87) |
|  | Respiratory/thoracic | 1.81 (0.93–3.54) | 2.21 (1.08–4.53) |
|  | Other | 0.94 (0.58–1.51) | 0.85 (0.48–1.51) |
|  |  |  |  |
| Cancer region males | Digestive/ gastrointestinal | Reference | Reference |
|  | Head and neck | 0.70 (0.48–1.03) | 0.52 (0.35–0.75) |
|  | Haematological/blood | 0.47 (0.27–0.81) | 1.18 (0.63–2.22) |
|  | Respiratory/thoracic | 1.22 (0.76–1.96) | 1.80 (1.06–3.04) |
|  | Other | 0.57 (0.39–0.84) | 0.66 (0.44–1.00) |
|  |  |  |  |
| Cancer stage | I | Reference | Reference |
|  | II | 1.25 (0.99–1.58) | 1.17 (0.82–1.65) |
|  | III | 1.20 (0.94–1.53) | 2.29 (1.65–3.18) |
|  | IV | 1.43 (1.05–1.95) | 4.59 (3.14–6.71) |
|  | None (haematological cancers) | 0.95 (0.69–1.29) | 2.86 (1.99–4.11) |
|  |  |  |  |
| EQ5D score (per 0.1 decrement) |  | 1.08 (1.04–1.12) | 1.20 (1.15–1.25) |
